# Iso-Osmolar Iodixanol Induces Less Increase in Circulating Endothelial Microparticles* In Vivo* and Less Endothelial Apoptosis* In Vitro* Compared with Low-Osmolar Iohexol

**DOI:** 10.1155/2018/8303609

**Published:** 2018-04-10

**Authors:** Beijian Zhang, Yi Zhang, Bo Liu, Lu Fang, Yigang Li, Shu Meng

**Affiliations:** ^1^Department of Cardiology, Xinhua Hospital, Shanghai Jiaotong University School of Medicine, Shanghai, China; ^2^Haematopoiesis and Leukocyte Biology Laboratory, Baker Heart and Diabetes Research Institute, Melbourne, VIC, Australia

## Abstract

**Background and Aims:**

There is no consensus on whether iodixanol is superior to iohexol. This study aimed to compare the effects of iodixanol and iohexol on circulating endothelial microparticles (EMPs) in stable coronary artery disease (CAD) patients with diabetes mellitus (DM), and also their cytotoxic effects on human umbilical vein endothelial cells (HUVECs)* in vitro*.

**Methods:**

100 CAD patients with DM were randomly assigned to receive iso-osmolar contrast medium iodixanol (group I) or low-osmolar iohexol (group II) during coronary angioplasty. An additional 49 CAD patients without DM receiving iohexol were recruited as group III. Circulating CD31^+^/CD41a^−^ EMPs, CD62E^+^ EMPs, and CD31^+^/CD41a^+^ platelet microparticles (PMPs) were determined by flow cytometry.* In vitro*, the cytotoxic effects of iodixanol and iohexol on HUVECs were determined.

**Results:**

Circulating CD31^+^/CD41a^−^ EMPs and PMPs were significantly increased after angioplasty in all 3 groups, while CD62E^+^ EMPs significantly decreased in group I. CD31^+^/CD41a^−^ EMPs and PMPs were significantly higher in group II than group I or III.* In vitro*, both contrast media induced EMP release and inhibited the viability and induced apoptosis of HUVECs, as well as increasing Bax and cleaved caspase-3 and decreasing Bcl-2. The above effects were less evident in iodixanol than in iohexol.

**Conclusions:**

Compared with iohexol, iodixanol induces less release of EMPs in both CAD patients with DM during angioplasty and* in vitro* HUVEC culture, which is associated with less pronounced proapoptotic effects of iodixanol on HUVECs.

**Clinical Study Registration Number:**

This study is registered with ChiCTR-TRC-14005183.

## 1. Introduction

Endothelial dysfunction, characterized by the loss or dysregulation of the endothelium's normal hemostatic mechanisms and its acquisition of proinflammatory and prothrombotic phenotypes, plays a critical role in the initiation and development of atherosclerosis [1–3]. Endothelial dysfunction is one of the important complications of intravascular administration of contrast media (CMs) [4–6], in the process of diagnostic angiography and percutaneous coronary intervention (PCI). Hyperosmolality, high viscosity, and chemotoxicity of CMs lead to dehydration and shrinking of endothelial cells. Endothelial damage due to exposure to CMs promotes atherosclerosis and precedes acute ischemic events or thromboembolic events [7].

Accumulating evidence has demonstrated that endothelial microparticles (EMPs) are emerging as a useful biomarker of endothelial dysfunction and/or injury, which are released from activated or apoptotic endothelial cells [8–10]. The circulating EMPs are significantly elevated in many pathological processes of vascular endothelial injury, such as coronary artery disease (CAD), diabetes mellitus (DM), stroke, and thrombosis [11–13], which suggests that EMPs may be involved in multiple pathophysiological processes in the body, such as hypoxia, hypoxia-related oxidative stress, thrombosis, inflammation, and atherogenesis [14]. A number of studies have also reported that EMPs are a robust predictor of all-causes and main cardiovascular mortality and cardiovascular events [9, 15, 16].

A lot of clinical evidence has shown that patients who received nonionic low-osmolar contrast media (LOCM, but still higher relative to plasma) or nonionic iso-osmolar contrast media (IOCM, iso-osmolar to plasma) have an advantageous outcome profile over ionic high-osmolar contrast media (HOCM) in diagnostic and interventional vascular procedures [17–19], leading to the replacement of HOCM by LOCM and IOCM in clinical practice. However, there is no consensus on whether nonionic dimers isomolar iodixanol is superior to nonionic monomer low-isomolar iohexol. Some studies suggest that iodixanol is associated with a reduction of adverse cardiac events and improved renal safety when compared to iohexol [20, 21]. However, other studies report conflicting results [22, 23] and raised doubts about the clinical advantages of iodixanol over iohexol.

In the present study, we aimed to compare levels of circulating EMPs in stable CAD patients with DM receiving iodixanol and in those receiving iohexol during selective PCI and to compare the effects of iodixanol and iohexol on the induction of endothelial cell apoptosis and the release EMPs* in vitro*.

## 2. Patients and Methods

### 2.1. Patients and Control Subjects

In this prospective, randomized controlled, double-blind trial, 100 stable CAD patients with DM in our hospital from January 2015 to June 2016 were enrolled and were randomly divided into group I (*n* = 50) and group II (*n* = 50) by random number table. An additional subgroup of 50 coronary heart disease patients without diabetes (group III) was recruited as control. One patient of group III refused the informed consent and was excluded, so the final number of group III was 49. All these patients underwent selective PCI, and iodixanol (Visipaque™ 320 mg I/mL) was used in group I, while iohexol (Omnipaque™ 300 mg I/mL) was used in groups II and III. The study complied with the Declaration of Helsinki and was registered (ChiCTR-TRC-14005183). The research program was approved by Xinhua Hospital Ethics Committee Affiliated to Shanghai Jiaotong University School of Medicine (XHEC-C-2014-045-2). All patients provided written informed consent.

Patients with stable CAD and with or without DM were eligible for the study, if they underwent selective PCI. Exclusion criteria included (1) age < 18 years or >80 years, (2) the use of any CM in the previous 3 months, (3) cardiovascular events or surgery in the past 3 months, (4) chronic kidney disease at stage 2 or above, (5) acute coronary syndrome, (6) acute or chronic infection, trauma, active rheumatism, or elevated C-reactive protein, (7) ejection fraction < 50%, or accompanied instable hemodynamics, (8) other diseases that may affect circulating EMPs, for example, tumor, thyroid disorders, tuberculosis, using hormones, or systemic lupus erythematosus, and (9) unlikely cooperation in the study.

Baseline characteristics of study subjects were obtained, including age, gender, body mass index, hypertension, DM, smoking history, family history of coronary heart disease, ejection fraction, laboratory parameters, and PCI-related parameters.

### 2.2. Randomization and Blinding

A random number table including 100 random numbers was generated by SPSS 23.0. Stable CAD patients with DM (*n* = 100) were randomly assigned to iodixanol group (group I) and iohexol group (group II) by a researcher independent of this study, at 1 : 1 ratio by the random number table (simple randomization). 49 stable CAD patients without DM receiving iohexol served as control (group III). Both researchers and participants were blinded to research groups. Each participant was allocated a code number relating to a CM, and no investigators had access to the key. The first unblinding was performed when all the data were obtained, and the second unblinding took place after all the statistical analysis was complete.

### 2.3. Blood Sampling and Preparation

An arterial blood sample (5 mL) was collected via radial artery sheath into 3.2% trisodium citrate vacutainer (Becton Dickinson, San Jose, CA) prior to CM injection and immediately following PCI. Erythrocytes, leukocytes, and platelets in the blood samples were removed by gradient centrifugation as mentioned previously [24]. Briefly, the blood was centrifuged at 160*g* for 10 minutes to prepare platelet-rich plasma and then centrifuged for 6 min at 1000*g* to prepare platelet-poor plasma. The plasma was stored at −80°C until further analysis [25].

### 2.4. Measurement of EMPs and PMPs by Flow Cytometry

Before flow cytometry, the plasma was thawed and incubated with fluorescent antibodies [25]. Briefly, a volume of 500 *μ*L of thawed plasma was centrifuged for 5 min at 16,000*g* at 4°C to remove residual platelet and debris. The top 450 *μ*L of plasma was transferred to a new tube and centrifuged for 30 min at 16,000*g* at 4°C to concentrate microparticles. The top 250 *μ*L of plasma was removed and the remaining 200 *μ*L was used for incubation with fluorescent antibodies for FCM.

The remaining 200 *μ*L of plasma was transferred to a TruCount tube preloaded with fluorescent bead (served as calculation reference) lyophilized pellets (Becton Dickinson Biosciences, San Jose, CA, USA, Cat#340334) and 3 *μ*L of each of CD31-AF488, CD41a-APC, and CD62E-PE (Becton Dickinson Biosciences, San Jose, CA, USA) was added to the tube and then incubated at 4°C for 30 min in the dark. The AF488-, APC- and PE-conjugated isotype control antibodies were used as control. Calibration beads (size in 0.2 *μ*m and 1.0 *μ*m, Molecular Probes, Eugene, Oregon, USA) served as size reference.

Acquisition of EMPs and PMPs was performed using a CytoFLEX S Flow Cytometer (Beckman Coulter, S.Kraemer Boulevard Brea, CA, USA). Analysis of EMPs and PMPs was operated at low flow-rate setting, and the light scatter and fluorescent was set at log model. Events between 0.1 *μ*m and 1.0 *μ*m in size on FS-SS graph were gated as EMPs or PMPs. EMPs were defined as CD31^+^/CD41a^−^ or CD62E^+^, while PMPs were defined as CD31^+^/CD41a^+^. Data of 10,000 events were obtained and analyzed using CytExpert (Version 2.0, Beckman Coulter).

The absolute number of EMP and PMP was calculated by the formula (number of EMP or PMP region × total number of beads per tube)/(number of beads collected × tested volume (200 *μ*L)). The total number of beads per tube was provided by the manufacturer. The results were expressed as number of CD31^+^/CD41a^−^ EMP, CD62E^+^ EMP, and CD31^+^/CD41a^+^ PMP per *μ*L of plasma.

### 2.5. Cell Line and Cell Culture

The human umbilical vein endothelial cells (HUVECs) were used in the study to determine whether CMs induced apoptosis of EC* in vitro*. The HUVEC was obtained from the American Type Culture Collection (ATCC, Manassas, Virginia, USA) and was cultured with RPMI 1640 medium (Gibco, USA) with 10% fetal bovine serum (Gibco, USA). The cells were incubated in a humid incubator with 5% CO_2_ at 37°C. The subsequent experiments were performed when the cell confluence reached 70% to 80%.

### 2.6. Cell Proliferation/Cytotoxicity Assay

The Cell Counting Kit-8 (CCK-8) assay was performed to assess cell proliferation/cytotoxicity and was carried out according to the manufacturer's instructions. Briefly, 8 × 10^3^ cells/well were seeded in a 96-well plate and incubated overnight. The drug concentrations of 4 vol%, 10 vol% and 20 vol% of iodixanol (Visipaque 320 mg I/mL) and iohexol (Omnipaque 300 mg I/mL) were used, in an attempt to approximate the relative concentrations of contrast media in blood that might occur during the bolus-injection and circulation-diluted phases of drug administration. Complete growth medium was used as control in the study unless otherwise specified. At 0 h, 1 h, 2 h, 3 h, 4 h, 5 h, 6 h, 12 h, and 24 h after stimulation, 10 *μ*L of CCK-8 (5 mg/mL, Beyotime Biotechnology, China) was added to each well and the absorbance at 450 nm was determined by using a Quant microplate reader (BioTek), after incubating for 2 h. Each treatment was set in triplicate and the assays were repeated at least three times.

### 2.7. Flow Cytometry to Determine Apoptosis

Briefly, 1 × 10^6^ HUVEC cells/well were seeded in a 6-well plate and incubated overnight. After stimulation by 4 vol%, 10 vol%, 20 vol% of iodixanol or iohexol for 4 h, the HUVECs were collected carefully after digestion with trypsin enzyme without EDTA. Then the cells were resuspended in 300 *μ*L of Annexin V binding buffer and incubated with 4 *μ*L each of Annexin V-FITC and propidium (PI)-PE (Becton Dickinson, USA) for 15 min at room temperature in the dark. Then the apoptosis of HUVECs was determined by a BD FACSCanto II flow cytometry (Becton Dickinson, USA).

### 2.8. Hoechst Staining

To further demonstrate the apoptosis of HUVECs, after treatment with 20 vol% of iodixanol or iohexol for 4 h, the cells were stained with 0.5 mL of Hoechst-33258 (Beyotime Biotechnology, China) for 5 min at room temperature in the dark. The apoptotic cells were observed using a fluorescence microscopy (Olympus BX51). The nucleus of a normal cell was normal blue, but the nucleus of an apoptotic cell was light blue, accompanied by chromatin condensation and fragmentation.

### 2.9. Western Blotting

After treatment with 20 vol% of iodixanol or iohexol for 4 h, 5 × 10^6^ HUVECs were harvested and lysed for 20 min on ice in 100 *μ*L of RIPA buffer (Beyotime Biotechnology, China) supplemented with 1 *μ*L of PMSF (Beyotime Biotechnology, China). 10 *μ*L of total cellular protein was separated by SDS-PAGE and transferred to PVDF membranes. Membranes were probed with primary antibodies: *β*-actin (1 : 1000, Beyotime Biotechnology, China), Bcl-2, Bax, caspase-3, and cleaved caspase-3 (all in 1 : 1000, from Cell Signaling Technology, USA) at 4°C overnight and then incubated with respective secondary antibodies at room temperature for 1 h. The signals were detected via Enhanced Chemiluminescence Reaction (ECL+, Millipore, USA) and ChemiDocXRS+ (Bio-Rad). The density of each blot was determined by Image Lab 3.0 software (Bio-Rad). Each immunoblotting was repeated three times.

### 2.10. Flow Cytometry to Determine EMPs

1 × 10^6^ cells/well were seeded in a 6-well plate and incubated overnight. The cells were stimulated by 4 vol%, 10 vol%, and 20 vol% of iodixanol or iohexol, and the same amount of complete growth medium was used as control. After 4 h, 2 mL of supernatant was collected and centrifuged for 15 min at 1500*g* at 4°C to remove cell debris [26]. The top 1800 *μ*L supernatant was transferred to a new tube and centrifuged for 30 min at 16,000*g* at 4°C to concentrate microparticles. The top 1500 *μ*L of supernatant was removed and the remaining 300 *μ*L was used to be incubated with fluorescent antibodies for flow cytometry. The processes of incubating antibodies and flow cytometry were described as above.

### 2.11. Statistical Analysis

Data analysis was performed by SPSS 23.0 (IBM for windows). Normal variates were represented by mean ± standard deviation (SD), and unpaired *t*-test or one-way analysis of variance (ANOVA) was performed to determine the differences. Since Kolmogorov-Smirnov test showed that EMPs and PMPs were nonnormally distributed, log-transformed data were used for analysis. Nonnormal data were presented as median and interquartile range (IQR) and analyzed by Kruskal-Wallis *H* test. Categorical variables were expressed as number of cases and percentage, and the differences were compared with the chi-square test. A two-tailed *P* < 0.05 indicates statistical significance.

## 3. Results

### 3.1. Patients' Baseline Characteristics

The baseline characteristics of these participants were reported in Table 1. The demographic data were comparable among three groups. Fasting blood glucose, blood urea nitrogen, and creatine were different among 3 group, and post hoc analysis showed that blood glucose was lower in group III compared to groups I and II. Pairwise comparisons showed that the differences in both urea and creatinine between group I and group II were not significant (urea: 5.3 (4.6, 6.5) versus 6.0 (5.0, 7.6), *P* = 0.333; creatine: 66.5 (59.8, 76.3) versus 72.9 (62.5, 89.4), *P* = 0.066). The levels of urea and creatine in these three groups were in the normal range. There were no significant correlations between kidney function and microparticles (data not shown). The volume of CM was determined by the complexity of PCI procedures, age, and renal and heart function of patients. And the volume was recorded by a nurse after a PCI procedure was finished. 150 mL of contrast media was used in the majority of patients who underwent PCI procedures, while the minimum volume and the maximum volume were 50 mL and 250 mL, respectively. Since the volume of CM was nonnormally distributed and was expressed as median and quartiles, so the median volume of CM was 150 mL in all three groups.

### 3.2. The Release of EMPs and PMPs after Exposure to CMs

**Figure 1:**
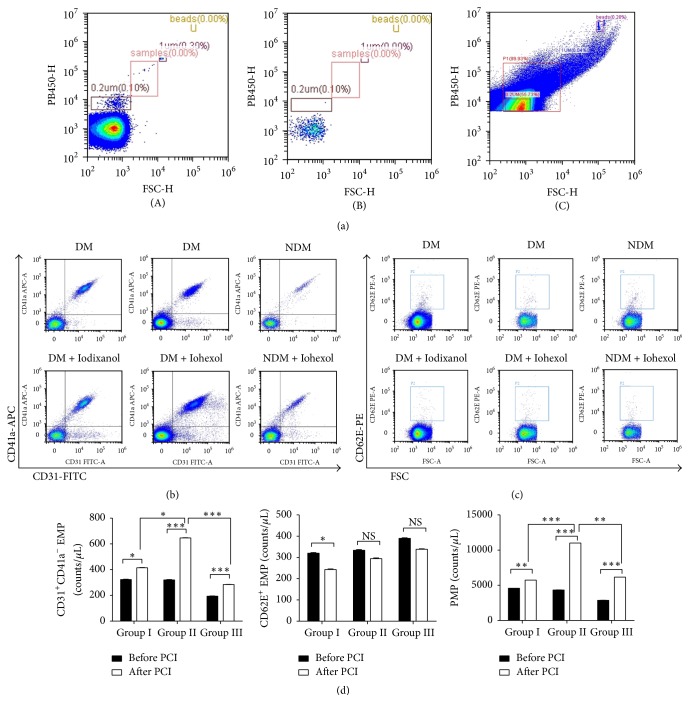
Effects of iodixanol and iohexol on circulating levels of CD31^+^/CD41a^−^ EMPs, CD62E^+^ EMPs, and PMPs. Gating of microparticles (a). The flow cytometry was referenced by calibration beads (size in 0.2 *μ*m and 1.0 *μ*m) (A). Acquisition of PBS, which was filtered by a filter in 0.22 *μ*m (B). Events between 0.1 *μ*m and 1.0 *μ*m in size on FS-SS graph were gated as microparticles (C). Measurement of plasma EMPs and PMPs at baseline and after injection of iodixanol or iohexol during PCI in patients with stable coronary artery disease (b–d). Staining of microparticles with CD31-FITC and CD41a-APC (b) and CD62E-PE (c), and results of CD31^+^CD41a^−^ EMP, CD62E^+^ EMP, and PMPs in 3 groups (d). The data were expressed as mean ± SD. DM: diabetes mellitus; NDM: non-diabetes mellitus; NS: nonsignificant; PCI: percutaneous coronary intervention. ^*∗*^*P* < 0.05, ^*∗∗*^*P* < 0.01, and ^*∗∗∗*^*P* < 0.001.

The counts of EMPs and PMPs were shown in Figure 1. At baseline, CD31^+^/CD41a^−^ EMPs and CD31^+^/CD41a^+^ PMPs in group III (patients without DM) were significantly lower than those in group I and group II, but there were no significant differences in CD31^+^/CD41a^−^ EMPs and PMPs between group I and group II. After exposure to CMs, CD31^+^/CD41a^−^ EMPs and PMPs were significantly increased in all of these three groups. Post hoc multiple comparisons indicated that the levels of CD31^+^/CD41a^−^ EMPs and PMPs were lower in iodixanol (group I) compared to iohexol (group II). When exposed to iohexol, stable CAD patients with DM (group II) were more likely to release more CD31^+^/CD41a^−^ EMPs and PMPs than patients without DM (group III) (Figures 1(b) and 1(d)).

The baseline levels of CD62E^+^ EMPs were comparable among three groups; however CD62E^+^ EMPs were decreased after exposure to iodixanol (Figures 1(c) and 1(d)). After exposure to CMs, the ratio of CD62E^+^/CD31^+^ EMP populations was <1.0, suggesting that EMPs are released from EC apoptosis instead of EC activation [27].

### 3.3. The Influence of CMs on Cell Proliferation/Cytotoxicity

**Figure 2:**
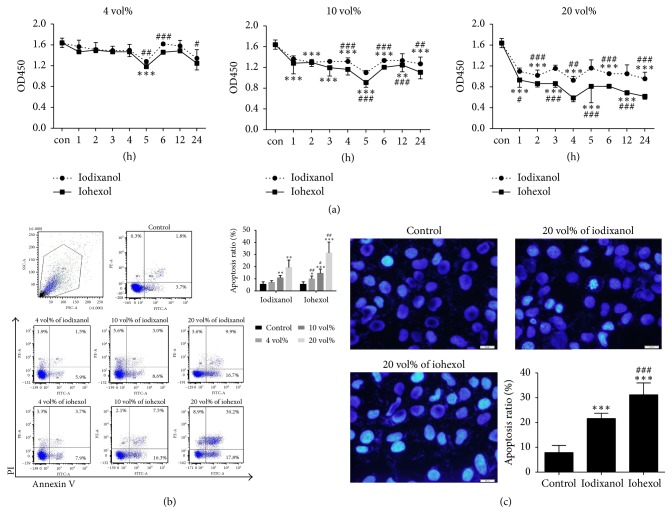
The effects of iodixanol and iohexol on viability and apoptosis of HUVECs. (a) HUVECs were stimulated with iodixanol or iohexol at different concentrations and time points and then cell proliferation/cytotoxicity assay was performed using CCK-8 assay. (b) HUVECs were treated with iodixanol or iohexol at different concentrations for 4 h and cell apoptosis was determined by Annexin V and PI staining. (c) HUVECs were treated with 20 vol% of iodixanol or iohexol for 4 h and stained with Hoechst. Reference bar size in 20 *μ*m. Magnification × 400 folds. The data were expressed as mean ± SD (*n* = 9). Complete growth medium served as control. ^*∗*^*P* < 0.05, ^*∗∗*^*P* < 0.01, and ^*∗∗∗*^*P* < 0.001 versus control; ^#^*P* < 0.05, ^##^*P* < 0.01, and ^###^*P* < 0.001 versus iodixanol.

Next, in* in vitro* HUVEC culture, we first examined the effects of different concentrations of iodixanol and iohexol on HUVEC proliferation/cytotoxicity after stimulation for 1 h, 2 h, 3 h, 4 h, 5 h, 6 h, 12 h, and 24 h (Figure 2(a)). At the concentration of 4 vol%, the viable cell number at 5 h was significantly decreased in both iohexol and iodixanol group compared with the control group and cell viability was significantly decreased in iohexol compared to iodixanol at 5 h, 6 h, and 24 h (Figure 2(a)). At the concentration of 10 vol%, both CMs significantly decreased cell viability at all time points, while the differences in cell viability between iohexol and iodixanol reached significance starting from 4 h. When the concentration of CMs increased to 20 vol%, the reduction of cell viability was significant at all time points compared to the control, with the greatest reduction at 4 h. Iohexol more significantly reduced viable cell number at all time points compared to iodixanol. Based on these results, the time point of 4 h was chosen in the subsequent experiments.

### 3.4. The Apoptosis of HUVECs Detected by Annexin V Staining

To investigate whether CMs induced the apoptosis of HUVECs, the cells were stimulated by 4 vol%, 10 vol%, and 20 vol% of iodixanol or iohexol for 4 h and apoptosis was detected by Annexin V-FITC and PI-PE staining. Both iodixanol and iohexol induced apoptosis of HUVECs in a dose-dependent manner (Figure 2(b)). All of three concentrations of iodixanol induced significantly less apoptosis compared to the same concentration of iohexol (Figure 2(b)).

### 3.5. The Apoptosis Cells Detected by Hoechst 33258 Staining

The apoptosis of HUVECs was also measured by the nucleus dye Hoechst 33258 staining. After exposure to 20 vol% of iodixanol or iohexol for 4 h, the HUVECs were stained with Hoechst 33258 and the apoptotic cells were counted using a fluorescence microscopy. Both groups had significantly higher apoptosis rates than the control group (21.6%  ±  2.1% and 31.2%  ±  4.8% versus 7.9%  ±  2.8%, respectively, both *P* < 0.001), but iodixanol group had significantly fewer apoptosis cells compared with iohexol group (Figure 2(c)).

### 3.6. The CMs Induced Apoptosis through Bcl-2/Bax-Caspase-3 Pathway

**Figure 3:**
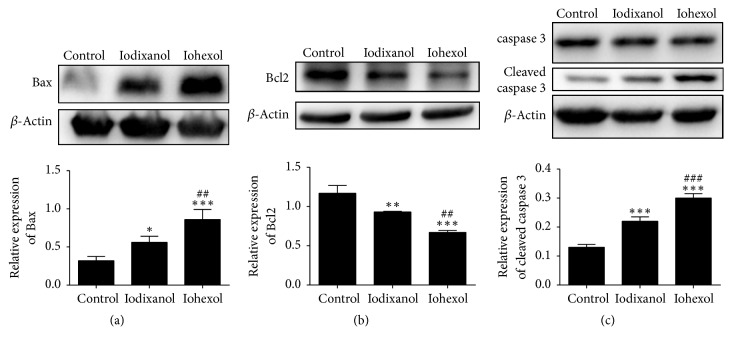
The effects of contrast media on apoptosis-related proteins in HUVECs. HUVECs were treated with 20 vol% of iodixanol or iohexol for 4 h and expression of Bcl-2 (a), Bax (b), caspase 3, and cleaved caspase-3 (c) was measured by Western blotting. These experiments were repeated at least 3 times. Data represents mean ± SD. Complete growth medium served as control. ^*∗*^*P* < 0.05, ^*∗∗*^*P* < 0.01, and ^*∗∗∗*^*P* < 0.001 versus control; ^#^*P* < 0.05, ^##^*P* < 0.01 and ^###^*P* < 0.001 versus iodixanol.

We further measured apoptosis-related proteins of HUVECs in response to CMs. After stimulation by 20 vol% of iodixanol or iohexol for 4 h, the expression of Bcl-2, Bax, caspase-3, and cleaved caspase-3 was determined by Western blotting. Compared to the control, the level of antiapoptotic Bcl-2 significantly decreased, while the levels of Bax and cleaved caspase-3 significantly increased after exposure to CMs (Figure 3). Compared to the iohexol group, the level of antiapoptotic Bcl-2 was significantly higher, while the levels of Bax and cleaved caspase-3 were significantly lower in the iodixanol group (Figure 3).

### 3.7. The Release of EMPs from HUVECs in Response to CMs* In Vitro*

**Figure 4:**
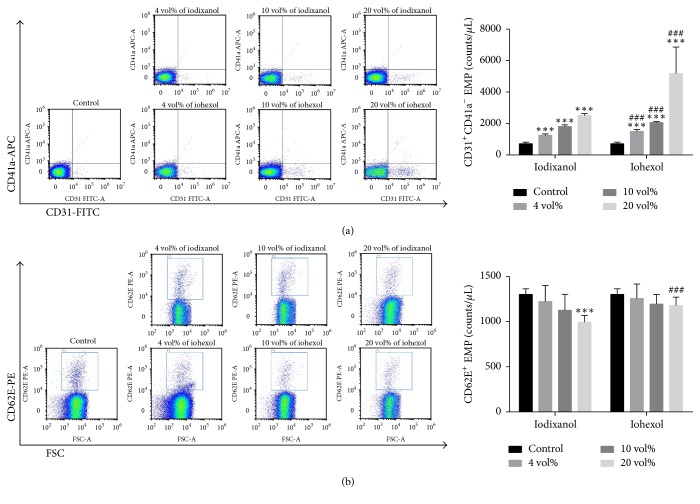
The induction of EMP release from HUVECs by iodixanol or iohexol. HUVECs were treated with different concentrations of iodixanol or iohexol for 4 h and CD31^+^/CD41a^−^ EMPs (a) and CD62E^+^ EMPs (b) were measured by flow cytometry. The data were expressed as mean ± SD (*n* = 9). Complete growth medium served as control. ^*∗∗∗*^*P* < 0.001 versus control; ^###^*P* < 0.001 versus iodixanol.

To confirm that EMPs are released from apoptotic cells induced by CMs* in vitro*, we measured CD31^+^CD41a^−^ EMPs and CD62E^+^ EMPs in the supernatant by flow cytometry. After exposure to either iodixanol or iohexol for 4 h, the release of CD31^+^CD41a^−^ EMPs increased significantly in a dose-dependent manner (Figure 4(a)). The counts of CD31^+^CD41a^−^ EMPs were significantly lower in iodixanol group than those of iohexol group, at all 3 concentrations. Interestingly, the level of CD62E^+^ EMPs tended to decrease in response to either iodixanol or iohexol, and its reduction reached statistical significance for the comparison between 20 vol% of iodixanol group and the control group (Figure 4(b)). CD62E^+^ EMPs were significantly lower in 20 vol% iodixanol group, compared to 20 vol% iohexol group.

## 4. Discussion

Conflicting data are generated with regard to whether iodixanol is superior to iohexol when used in diagnostic and interventional procedures. In the present study, we compared the effects of these two CMs on endothelial dysfunction* in vivo* and* in vitro*. First, we demonstrated that circulating EMPs levels significantly increased in patients with stable CAD after receiving iodixanol or iohexol during PCI procedure, and iodixanol induced less EMPs release than iohexol in patients with stable CAD. In* in vitro *HUVEC culture, we demonstrated that both iohexol and iodixanol significantly induced EMP release from HUVECs, inhibited proliferation, and increased apoptosis of HUVECs through Bcl-2/Bax-caspase-3 signal pathway, but iodixanol is less cell toxic than iohexol. In addition, iohexol causes more marked increase in CD31^+^/CD41a^−^ EMPs and PMPs in stable CAD patients with DM than in those without DM, indicating that diabetic patients are more sensitive to iohexol. Taken together, iodixanol causes less damage to endothelial cells compared to iohexol in CAD patients with DM.

First, we confirmed the effects of both iodixanol and iohexol on endothelial dysfunction. After exposure to iodixanol or iohexol, circulating CD31^+^/CD41a^−^ EMPs and PMPs increased significantly in patients with stable CAD compared to the baseline. The endothelial damage and thrombogenicity are considered to be risks of CM use. Previous studies reveal that water deprivation [28], oxidative stress [29], vasoconstriction [30], and ischemic damage [31] are the most prominent predisposing factors of the toxic effect of CMs on renal tubules* in vivo* and may be the underling mechanism of contrast-induced acute kidney injury. Moreover, both iodixanol and iohexol induced release of EMPs from HUVECs and inhibited proliferation/viability and induced apoptosis of HUVECs* in vitro* not only in a time-dependent manner but also in a dose-dependent manner.

IOCM iodixanol is a nonionic dimer, which is an emerging category of CMs. Documented literatures have reported that iodixanol has less cardiovascular events in the process of cardioangiography, compared to ionic low-osmolar ioxaglate [17] and nonionic low-osmolar iohexol [20]. In the present study, we found that the counts of plasma EMPs and PMPs after PCI were significantly lower in stable CAD patients with DM receiving iodixanol than those receiving iohexol, indicating that iodixanol has less impact on endothelial damage and platelet activation. Consistently,* in vitro*, iodixanol induced less release of EMPs by HUVECs than iohexol, which is associated with less pronounced effects of iodixanol on inducing cell apoptosis and reducing cell viability. The dysfunction of endothelium potentiates the procoagulant status [32]. Moreover, elevated EMPs and PMPs per se not only are associated with thrombosis and inflammatory state predisposed to atherosclerosis and CAD development, but also increase blood viscosity, which exacerbates the state of hypercoagulability and increases cardiovascular events following the endothelium damage and platelet activation [9, 33]. Hence, iodixanol may be a better choice than iohexol in the procedure of coronary angioplasty, especially for CAD patients with DM as demonstrated in this study, because it induces less endothelial damage and platelet activation, and less microparticles release.

Endothelial dysfunction [34] and platelet activation [35] are the common characteristics of diabetes. We found that stable CAD patients with DM had higher levels of CD31^+^/CD41a^−^ EMPs and PMPs than patients without DM at baseline, which is in agreement with previous studies [25]. Diabetes is a risk factor for higher subsequent revascularization rates, lower one-year survival [36], and more major adverse cardiac events (MACEs) after PCI [37]. In the present study, we found that the increment of CD31^+^/CD41a^−^ EMPs and PMPs after injection of iohexol was more in patients with DM than in those without DM, which indicates that diabetes patients are more sensitive to iohexol. This further supports that iohexol may be not a good choice for diabetes patients in the procedure of angiography and PCI.

The levels of CD62E^+^ EMPs at baseline are not significantly different between patients with DM and those without DM, which is in consistent with previous studies reporting that CD62E^+^ EMPs are normal in patients with the metabolic syndrome [38] and patients with type 2 DM [39] compared to healthy subjects. Interestingly, after exposure to CMs, a downward trend of CD62E^+^ EMPs was observed but the decrement reached statistical significance only in iodixanol group. As stated in the previous literature, the ratio of CD62E^+^/CD31^+^ EMPs ≤ 1.0 suggests endothelial apoptosis, while ≥10 suggests endothelial activation [27]. The ratio of CD62E^+^ EMPs/CD31^+^ EMPs was <1 after injection of CMs in our study, indicating that EMPs are released by apoptotic endothelial cells, which was confirmed by our further experiments* in vitro*.

Previous studies have shown that caspase-3 and Bcl2/Bax are involved in CMs induced apoptosis [40–42]. In the present study, we showed that proapoptotic Bax and cleaved caspase-3 increased while antiapoptotic Bcl-2 and the ratio of Bcl-2/Bax decreased after exposure to CMs, implying that CMs induced apoptosis of HUVECs at least partly via Bcl-2/Bax-caspase-3 signal pathway. The expression of Bax and cleaved caspase-3 was significantly decreased while the expression of Bcl-2 was higher in iodixanol group than in iohexol group, which explains the less proapoptotic effect of iodixanol than iohexol on HUVECs.

There are some limitations in the study. First, due to some difficulties, we were unable to measure microparticles immediately in freshly collected blood samples. It is known that microparticles may be affected by freezing and thawing process. However, our plasma samples were subjected to a single freeze-thaw cycle. Second, we did not collect follow up data on clinical end-points in this study. It would be interesting to correlate clinical end-point data with circulating levels of EMPs in patients receiving either iodixanol or iohexol. Therefore, although we conclude that iodixanol induces less release of EMPs than iohexol in this study, selection of iodixanol over iohexol in CAD patients with DM needs to be validated in future clinical studies.

## 5. Conclusion

IOCM iodixanol induces less of an increase in circulating EMPs and PMPs in stable CAD patients with diabetes compared with LOCM iohexol, when used in PCI procedure.* In vitro*, Iodixanol has less impact on the release of EMPs from HUVECs, which is associated with less pronounced cytotoxic and proapoptotic effects of iodixanol on HUVECs, compared with iohexol. In addition, injection of iohexol causes more marked increase in CD31^+^/CD41a^−^ EMPs and PMPs in patients with DM than in those without DM, indicating that diabetic patients are more sensitive to iohexol. Therefore, iodixanol may have advantage over iohexol in CAD patients with diabetes in the procedures of cardioangiography and coronary angioplasty.

## Figures and Tables

**Table 1:** Patients' baseline characteristics.

Variates	Group I	Group II	Group III	*P*
*n*	50	50	49	/
Male, *n* (%)	28 (56.0)	31 (62.0)	37 (75.5)	0.116
Age, years	68.6 ± 8.8	68.6 ± 9.4	68.5 ± 9.5	0.998
BMI, kg/m^2^	25.1 ± 3.0	24.6 ± 2.6	24.9 ± 3.1	0.767
HBP, *n* (%)	41 (82.0)	43 (86.0)	35 (71.4)	0.175
Smoking history, *n* (%)	18 (36.0)	17 (34.0)	24 (49.0)	0.256
Family history, *n* (%)	33 (66.0)	33 (66.0)	25 (51.0)	0.212
Total cholesterol, mmol/L	4.1 ± 1.2	3.9 ± 0.9	3.9 ± 0.9	0.571
Total triglyceride, mmol/L	1.3 ± 1.7	1.3 ± 1.7	1.3 ± 1.7	0.999
HDL-C, mmol/L	1.2 ± 1.3	1.2 ± 1.2	1.2 ± 1.2	0.695
LDL-C, mmol/L	2.3 ± 1.6	2.1 ± 1.3	2.0 ± 1.4	0.216
hsCRP, mg/L	1.3 ± 4.3	1.8 ± 5.4	1.3 ± 4.0	0.495
Fasting blood glucose, mmol/L	7.1 ± 1.4	7.2 ± 1.3	5.3 ± 1.2	<0.001
Blood urea nitrogen, mmol/L	5.3 (4.6, 6.5)	6.0 (5.0, 7.6)	5.1 (3.8, 6.1)	0.003
Creatinine, *µ*mol/L	66.5 (59.8, 76.3)	72.9 (62.5, 89.4)	73.0 (62.0, 85.0)	0.046
ALT, U/L	18.5 (13.0, 25.8)	17.5 (13.0, 25.0)	18.0 (14.0, 23.5)	0.694
AST, U/L	19.5 (17.0, 23.3)	19.0 (15.8, 23.3)	20.0 (17.5, 23.5)	0.209
CK-MB, ng/mL	1.9 (1.1, 3.2)	2.0 (1.3, 3.6)	1.8 (1.2, 3.8)	0.846
Troponin-T, ng/mL	0.01 (0.01, 0.05)	0.01 (0.00, 0.03)	0.01 (0.00, 0.04)	0.223
Lesion arteries, *n*	2.0 (2.0, 3.0)	3.0 (2.0, 4.0)	2.0 (1.0, 3.0)	0.151
Implanted stents, *n*	1.0 (1.0, 1.0)	1.0 (1.0, 2.0)	1.0 (1.0, 2.0)	0.388
Gensini Score	27.7 ± 2.0	28.1 ± 2.4	28.7 ± 1.9	0.970
Volume of contrast media, mL	150.0 (150.0, 150.0)	150.0 (150.0, 150.0)	150.0 (150.0,150.0)	0.570

Normal data are represented by mean ± SD, nonnormal variates are repressed as median (interquartile range), and categorical variables are expressed as percentage. BMI: body mass index; HBP: high blood pressure; HDL-C: high-density lipoprotein cholesterol; LDL-C: low-density lipoprotein cholesterol; hsCRP: high sensitivity C-reactive protein; ALT: alanine aminotransferase; AST: aspartate aminotransferase; CK-MB: creatine kinase isoenzyme MB.
